# Activation of Dendritic Cells in Tonsils Is Associated with CD8 T Cell Responses following Vaccination with Live Attenuated Classical Swine Fever Virus

**DOI:** 10.3390/ijms22168795

**Published:** 2021-08-16

**Authors:** Ferran Soldevila, Jane C. Edwards, Simon P. Graham, Helen R. Crooke, Dirk Werling, Falko Steinbach

**Affiliations:** 1 Virology Department, Animal and Plant Health Agency, Addlestone KT15 3NB, UK; ferransolde@gmail.com (F.S.); Jane.Edwards@pirbright.ac.uk (J.C.E.); simon.graham@pirbright.ac.uk (S.P.G.); Helen.Crooke@apha.gov.uk (H.R.C.); 2 The Pirbright Institute, Ash Road, Pirbright GU24 0NF, UK; 3 Department of Pathobiology and Population Sciences, The Royal Veterinary College, Hawkshead Lane, Hatfield, Hertfordshire AL9 7TA, UK; Dwerling@RVC.AC.UK; 4 School of Veterinary Medicine, University of Surrey, Guildford GU2 7AL, UK

**Keywords:** dendritic cells, macrophages, myeloid, palatine tonsil, pig, CSFV

## Abstract

Classical swine fever (CSF) is a highly contagious disease caused by the classical swine fever virus (CSFV). The live attenuated C-strain vaccine is highly efficacious, initiating protection within several days of delivery. The vaccine strain is detected in the tonsil early after inoculation, yet little is known of the role that tonsillar immune cells might play in initiating protection. Comparing the C-strain vaccine with the pathogenic CSFV Alfort-187 strain, changes in the myeloid cell compartment of the tonsil were observed. CSFV infection led to the emergence of an additional CD163^+^CD14^+^ cell population, which showed the highest levels of Alfort-187 and C-strain infection. There was also an increase in both the frequency and activation status (as shown by increased MHC-II expression) of the tonsillar conventional dendritic cells 1 (cDC1) in pigs inoculated with the C-strain. Notably, the activation of cDC1 cells coincided in time with the induction of a local CSFV-specific IFN-γ^+^ CD8 T cell response in C-strain vaccinated pigs, but not in pigs that received Alfort-187. Moreover, the frequency of CSFV-specific IFN-γ^+^ CD8 T cells was inversely correlated to the viral load in the tonsils of individual animals. Accordingly, we hypothesise that the activation of cDC1 is key in initiating local CSFV-specific CD8 T cell responses which curtail early virus replication and dissemination.

## 1. Introduction

Classical swine fever (CSF) is a contagious disease caused by the classical swine fever virus (CSFV; Pestivirus C), a virus belonging to the *Pestivirus genus* of the *Flaviviridae* family. Acute infection is characterised by pyrexia, anorexia and haemorrhages of the skin and mucosal surfaces. The infection is often fatal and death typically occurs within 2 to 4 weeks following exposure [1,2]. In the case of a CSFV outbreak, the trade of pork and pork produce is restricted and countries wishing to reclaim a disease-free status apply a stamping out and ‘pre-emptive’ eradication strategy [3] resulting in the culling of large numbers of pigs, economic loss and damage to the pig industry [4,5].

Live attenuated vaccines such as the C-strain are widely used to control CSF in areas where the virus is endemic [6]. C-strain based vaccines are safe and efficacious, providing pigs with rapid protection from challenges with highly virulent and genetically divergent CSFV strains [7,8]. However, for the purpose of discriminating infected and vaccinated animals (DIVA), alternative vaccines are required. DIVA vaccines would provide better support for control/eradication programmes for endemic regions, as well as reduce the requirements for stamping to contain outbreaks elsewhere. Accordingly, deciphering the success of C-strain vaccines remains of interest, not only for controlling CSFV, but also for the development of improved vaccines for other pig viruses.

The oronasal route is the main entry of CSFV into the host and the palatine tonsils are one of the early targets for viral replication [9]. In the tonsil, the virus displays a tropism for endothelial cells, macrophages (MØ) and dendritic cells (DCs) [10,11,12,13,14,15]. From there, the virus traffics through the lymph to other lymphoid tissues, eventually entering the blood stream [10,16,17]. The importance of the tonsil for the pathogenesis of CSFV infections is also evident from the tropism of C-strain vaccines, which even upon peripheral injection primarily target and persist in the tonsil [18]. Moreover, when applied intranasally, the C-strain infection is almost completely restricted to the tonsil and surrounding lymphoid tissues and viraemia is undetectable [19] or present only at low levels [20]. Accordingly, the immune reactions in the tonsils are crucial for the outcome of CSFV infection and vaccination [18,20].

The C-strain vaccination induces robust protection against CSFV challenge as early as 5 days post-vaccination [7,8], preceding a detectable neutralising antibody response. Several studies have demonstrated the emergence of an CSFV-specific CD8^+^ cytotoxic T lymphocyte (CTL) response in the blood 6–8 days following challenge of vaccinated pigs [7,21,22]. However, the exact mechanisms underlying the protection are still unclear, specifically given the lower CTL response in vaccinated pigs that had not received a sequential challenge [23].

DCs play a central role in initiating adaptive immune responses, inducing primary T cell responses and are likely important players in the immunological events following C-strain vaccination. The interaction of tonsillar myeloid cell populations with CSFV has received limited attention so far. Plasmacytoid DCs (pDC) produce high levels of IFN-α following CSFV infection [24], which correlates with the severity of infection and the degree of lymphopenia induced by CSFV [25,26]. In addition, tonsillar conventional DCs (cDCs; defined then as CD11R1^+^ CD172a^+^) were found to contain the virus, but their frequency did change during the course of infection [15]. Since IL-12 release was reduced in CSFV infected DC populations, it appears that CSFV infection can modulate DC responses [27]. Furthermore, while the C-strain *Riems* protects within five days post vaccination, CSFV-specific T cells responses were not detected in pigs before challenge [21]. An improved understanding of the immunological mechanisms involved in the rapid protection following the C-strain vaccination is imperative in the design of improved vaccination strategies.

In this study, the early effect of CSFV on myeloid cell populations in the tonsil was investigated by comparing the attenuated C-strain vaccine with the virulent Alfort-187 strain. By tracing both the dynamics of infection and the frequency of each of the populations of myeloid cells previously identified in the porcine tonsil [28], it was possible to demonstrate changes in the composition of the myeloid compartment and particularly the DC subpopulations. The results also support the notion that CD8 T cell responses are initiated within the first days of the C-strain vaccination, but not the virulent CSFV infection.

## 2. Results

### 2.1. CSFV Infection Is Restricted to Tonsils during the Early Time Points

**Figure 1:**
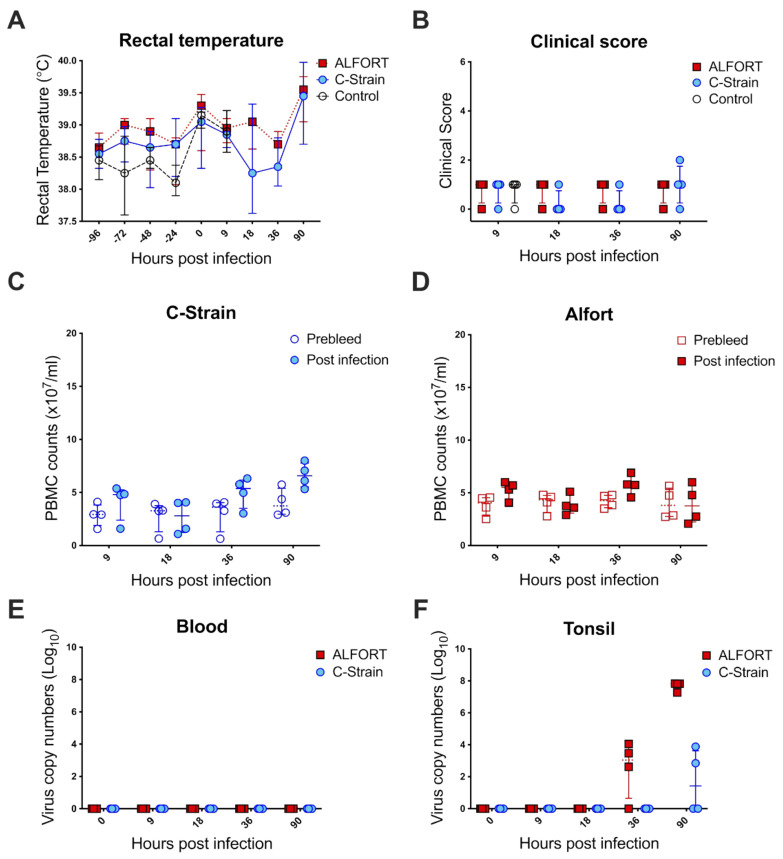
Clinical scores/rectal temperatures/PBMC counts/viral load comparison between CSFV C-strain and Alfort-187 inoculated pigs. A total of 36 pigs were intranasally infected with either mock, C-strain or Alfort. Pigs that received the mock vaccine were culled at 9 h post-inoculation, while 4 pigs from each group that received either the C-strain or Alfort-187 were culled at 9, 18, 36 and 90 h post-inoculation. Rectal temperatures (**A**) were taken every 24 h starting at 96 h prior to intranasal inoculation and up to and including 90 h following inoculation with either the C-strain (blue circles), Alfort-187 (red squares) or the mock virus (open circles). Results are expressed as the mean for each group and error bars represent one standard deviation (SD). The clinical score was determined (**B**) at each time point for each pig and again error bars represent one standard deviation (SD). For each time point, PBMCs were isolated from the respective groups, labelled for CD45 expression and counted using volumetric flow cytometry to assess leukopenia in (**C**) C-strain and (**D**) Alfort-187 infected animals. Data here were compared to the pre-bleed values from the same animals taken at 0 h. Plots show individual values for each pig sacrificed at each time point with the mean for each group. Error bars represent one standard deviation (SD). Finally, cells were isolated from tonsils and EDTA blood was collected from the 4 pigs sacrificed at each time point and the number of viral copy numbers determined (**E**) in the blood and (**F**) tonsil, respectively. The control animals are here put to 0 h to match the descriptive hours post-infection. Graphs show individual genome copy numbers for each pig in both tissues at different time points with the median ± interquartile range.

Over the 90 h assessed in this study, the pigs did not show any significant changes in rectal temperature (Figure 1A), nor were the clinical signs of the disease observed in any of the groups (Figure 1B). Leukopenia, a hallmark of clinical CSF, was not detected at such early time points (Figure 1C,D). Viremia was also absent in the blood (Figure 1C,D) as analyzed by RT-qPCR (Figure 1E). In the tonsil, however, the CSFV genome was found in animals exposed to the pathogenic CSFV Alfort-187 at 36 h in three of the four pigs (between 2.6 and 4.05 log_10_ copy numbers) and at 90 h post-infection in four of the four pigs and the number of viral copies had increased to 7.2–7.8 log_10_ copy numbers (Figure 1F). In C-strain infected animals, the virus was only detected in two of the four pigs after 90 h (2.8 and 3.8 log_10_ copy numbers) with the number of viral copies comparable to the Alfort-187 group at 36 h post-infection (Figure 1F).

### 2.2. CSFV Infection Leads to Changes in the Myeloid Cell Compartment and the C-Strain Alters the DC Composition

Given the described emergence of virus specific CD8^+^ T cells in the blood following C-strain vaccination and challenge [7,21,22] and the importance of DC in stimulating naïve T cells, we reasoned that the repertoire of DC in in the tonsil might be altered prior to detection of the T cell response and that these changes might differ to those seen following the pathogenic CSFV challenge. Accordingly, the frequency of the different myeloid cells populations was evaluated following CSFV C-strain or Alfort-187 infection as previously described in the uninfected porcine tonsil [28]. These populations included pDC (MHC-II^low^ CD172a^low/neg^ CD4^+^ CADM1^−^ CD14^−^ CD163^−^), cDC1 (MHC-II^high^ CD172a^low/neg^ CD4^−^ CADM1^high^ CD14^−^ CD163^−^), cDC2 (MHC-II^high^ CD172a^high^ CD4^−^ CADM1^low^ CD14^−^ CD163^−^), CD14^+^ monocytes (MHC-II^high^ CD172a^high^ CD4^−^ CADM1^low^ CD14^+^ CD163^−^) and CD163^+^ macrophages (MHC-II^high^ CD172a^high^ CD4^−^ CADM1^low^ CD14^−^ CD163^+^).

**Figure 2:**
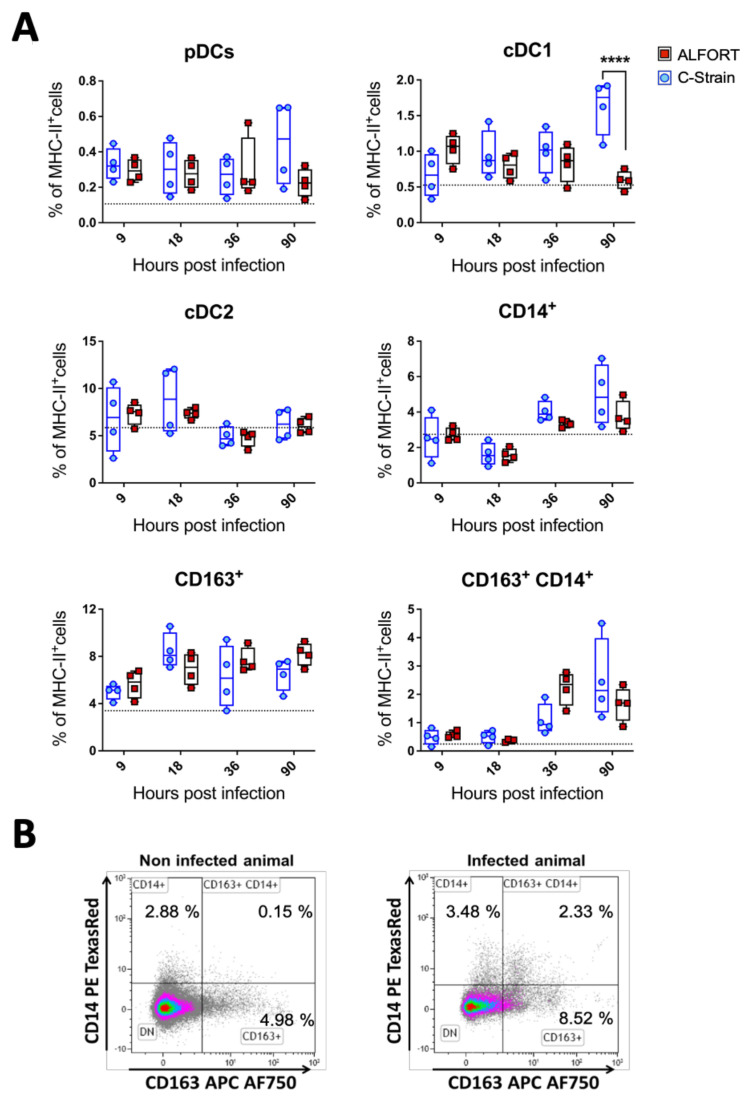
Myeloid cell frequencies in the tonsil during early stages of infection with CSFV C-strain and Alfort-187. (**A**) Percentage of each myeloid cell subset frequency from the total MHC-II^+^ positive cells. A total of 4 pigs at each time point from pigs infected with C-strain (blue open circles) or Alfort-187 (red filled circles) and a dashed black line corresponds to the median values obtained with the 4 mock infected animals. Boxes represent the 25/75% percentile of data with median and data shown. Statistical significances between C–Strain and Alfort-187 were evaluated by a two-way ANOVA with Bonferroni correction (**** *p* < 0.0001). (**B**) representative dot plots of a CD14^+^CD163^+^ population is shown using a representative sample 36 h post-infection with Alfort-187 and an uninfected control.

The frequency of pDC was elevated in all inoculated animals compared to the mock group, while there was an increase in C-strain infected animals towards the end of the study, but without statistical significance (Figure 2A). In C-strain vaccinated pigs, there was a significant increase in cDC1 cells which almost doubled in frequency by 90 h post-infection to 1.88% of MHC-II^+^ cells. Conversely, following Alfort-187 infection, cDC1 cells were slightly elevated after 9 h but then steadily declined in frequency to the same level as the mock group (0.65% of total MHC-II^+^ cells) (Figure 2A). cDC2 frequencies were not significantly altered across the study (Figure 2A), while the frequency of CD163^+^ macrophages was higher in both the virus-challenged groups compared to the mock-infected group (between 4 and 10.5% of MHC-II^+^ cells) and remained high for the duration of the study (Figure 2A). These differences were found not to be statistically signifcant. Similarly, the frequency of CD14^+^ monocytic cells also appeared to increase during the study but not in a statistically significant way (Figure 2A). Finally, during the analysis of the subpopulations, it became apparent that a double positive (CD14^+^CD163^+^) population of myeloid cells had emerged (Figure 2B). At 9 and 18 h post-infection with either strain of CSFV, this population was scarce but increased in frequency at 36 and 90 h post-challenge (up to 3–4% of MHC-II^+^ cells) (Figure 2A). Interestingly, the abundance of these cells seemed to peak at 36 h following Alfort-187 inoculation, compared to the C-strain, while in animals inoculated with the C-strain, these cells peaked at 90 h post inoculation but were not statistically significantly different from the controls.

### 2.3. Effect of CSFV Infection upon MHC-II Expression on Myeloid Cells

**Figure 3:**
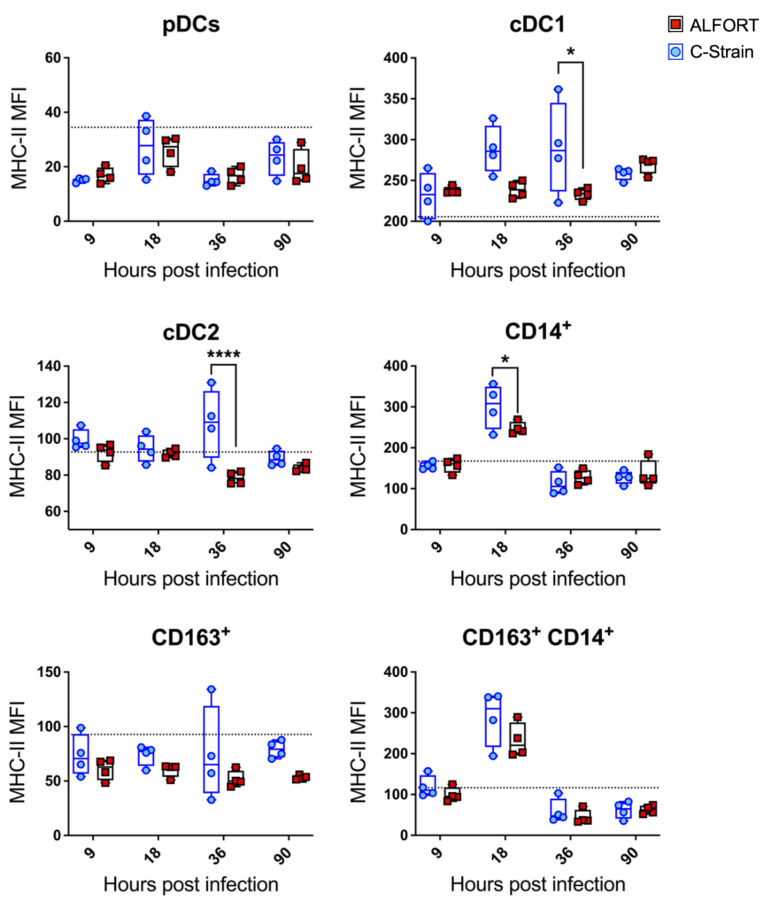
Differences in MHC-II expression levels in tonsillar myeloid cells following CSFV Alfort-187 and C-strain infection. The mean fluorescence intensity (MFI) of MHC-II expression associated with each of the tonsillar myeloid cell populations was evaluated at each time point following virus inoculation. Graphs show the MFI for 4 pigs culled at each time point for Alfort-187 (red squares), C-strain (blue circles) and the 9 h timepoint for the mock infected control animals (black dotted line). Boxes represent the 25/75% percentile of data with the median and data values shown. Statistical differences between C-strain and Alfort-187 were evaluated by a two-way ANOVA with Bonferroni correction (* *p* < 0.05, **** *p* < 0.0001).

Next, we assessed MHC class II expression as a measure of the ability of myeloid cells to function as antigen-presenting cells. Significant differences were observed in both the cDC1 and cDC2 populations and between the C-strain and Alfort-187 infections. Specifically, cDC1s upregulated the expression of MHC-II at 18 h and 36 h (*p* < 0.03) following C-strain infection relative to Alfort-187. Furthermore, higher levels of MHC-II were associated with cDC2 at 36 h post-infection in C-strain vaccinated pigs compared to Alfort-187 (*p* < 0.03) (Figure 3). Both CD14^+^ monocytic cells and the newly arising CD14^+^/CD163^+^ population seemed to have a peak expression of MHC-II at around 18 h, although this difference was only statistically significant for CD14^+^ cells between the two viral strains. No significant observations were made in pDC (which are relatively low in their MHC-II expression) and CD163^+^ macrophages, in which MHC-II expression remained relatively constant throughout the course of the study. Collectively, these results reveal an early activation of CD14^+^ and cDC cells in C-strain inoculated animals, which was specifically evident at 18 h and 36 h post-vaccination, respectively.

### 2.4. CSFV Has a Strong Tropism for the Newly Arising CD14^+^CD163^+^ Cells

**Figure 4:**
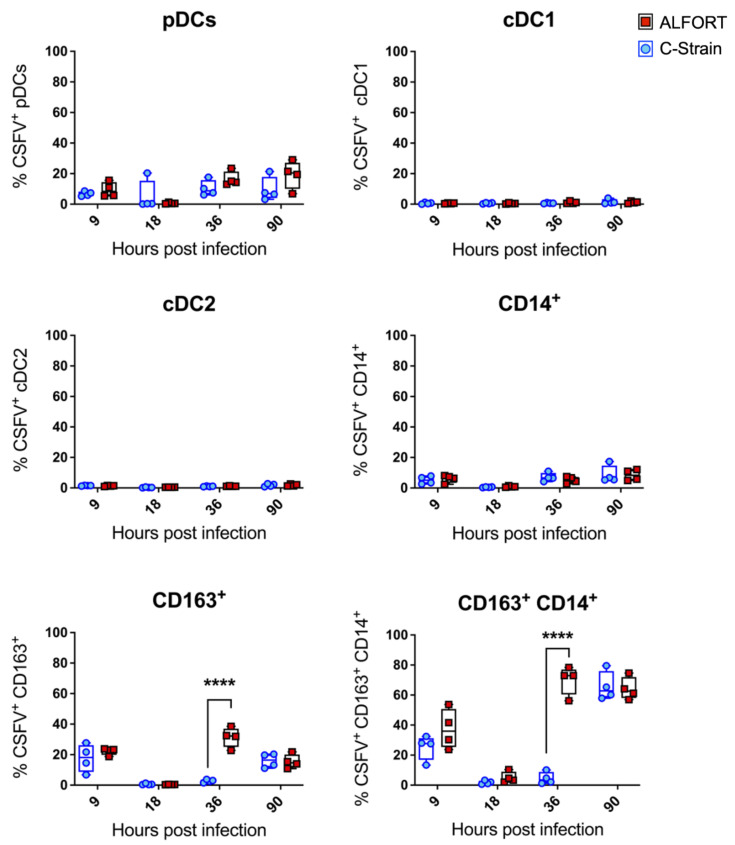
CSFV infection of tonsillar myeloid cell populations. Tonsillar myeloid cells were stained with antibodies to CD4, CD14, CD172a, CD163, CADM1 and MHC-II followed by intracellular staining for CSFV E2 using the monoclonal antibody WH303 to determine the infection status of each population at each time point. For each time point the median percentage of infected cells for the 4 pigs culled and data are shown. Boxes represent the 25/75% percentile. Statistical significances between the C–strain (blue circles) and Alfort-187 (red squares) were evaluated by a two-way ANOVA with Bonferroni correction (**** *p* < 0.0001).

Given the known tropism of CSFV for myeloid cells and the ability of the virus to modulate this [15,29,30], we next assessed the CSFV infection status of the myeloid cell populations by E2 intracellular staining (representative flow cytometry dot plots demonstrating E2 staning are shown in Supplementary Figure S1). Interestingly, neither the cDC1 nor the cDC2 cells demonstrated any susceptibility for CSFV at the timepoints examined (Figure 4). pDC and to an even lesser extent CD14^+^ monocytic cells, showed little susceptibility for CSFV infection, with a maximum of around 20% of these cell populations infected by the end of the experiment. The CD163^+^ macrophages also had a limited susceptibility, but here a significant difference between the Alfort-187 and C-strains could be observed, whereby the pathogenic Alfort-187 strain infected a larger proportion of macrophages at 36 h. The most susceptible population were the CD14^+^/CD163^+^ cells. Here, around 75% of the population stained positive for E2 at both 36 and 90 h for Alfort-187 and at 90 h for the C-strain. 

### 2.5. Detection of CSFV-Specific T Cell Responses in Tonsils of Inoculated Animals

**Figure 5:**
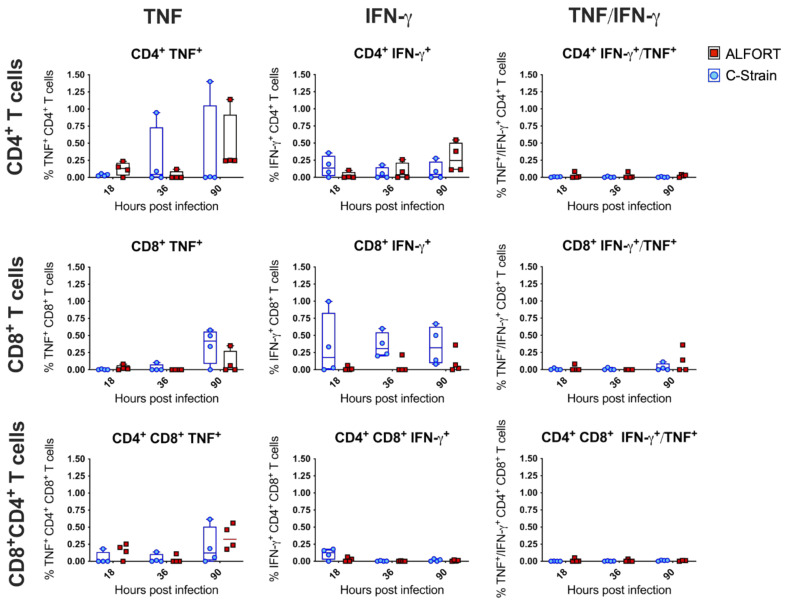
Evaluation of T cell associated IFN-γ and TNF-α responses from Alfort-187 and C-strain infected pigs in the tonsil. Porcine tonsil mononuclear cells were stimulated by the addition of CSFV Alfort-187. Following overnight culture, cells were stained and the cytokine expression associated with each of the T cell populations was evaluated by flow cytometry. Histograms show the mean IFN-γ and TNF-α secretion associated with each T cell population. Values and the medians were plotted; boxes represent the 25/75% precentile. Statistical significances were assessed by a two-way ANOVA with Bonferroni correction again.

Virus-specific, IFN-γ secreting CTLs have been shown to be present in the blood of C-strain vaccinated pigs following challenge, but not before 5 days post-vaccination, when protection by the C-strain is already achieved [22]. Given the alterations we have described in the myeloid cell compartment following the C-strain vaccination and the possibility that some of these cells may be equipped to serve as antigen-presenting cells, we attempted to detect the T cell responses arising locally in the tonsil. Cells were stained with antibodies to identify CD4^−^CD8α^hi^, CD4^+^CD8α^−^ and CD4^+^CD8α^+^ populations representing cytotoxic CD8, naïve CD4 helper and CD4 activated/memory populations, respectively [31] (the flow cytometric approach is shown in Supplementary Figure S2). CSFV-specific T cells expressing IFN-γ, TNF or both TNF and IFN-γ were detected (Figure 5). While none of the results, bar one, were statistically significant, some trends emerged. IFN-γ^+^ CD8^+^ T cell responses appeared greater at all timepoints after the C-strain vaccination compared to the Alfort-187 infection, with the highest differences observed at 36 h post-infection; however, no statistically significant differences were found (*p* < 0.0918) (Figure 5). Similarly, TNF^+^ CD8^+^ T cells seemed to arise at 90 h post-infection, particularly in the C-strain vaccinated animals. In all other cases where T cell activation was measured, the responses were similar across both viruses.

### 2.6. Correlation between DC Modulation and CSFV-Specific IFN-γ^+^ CD8^+^ T Cell Responses with Viral Load in the Porcine Palatine Tonsil

**Figure 6:**
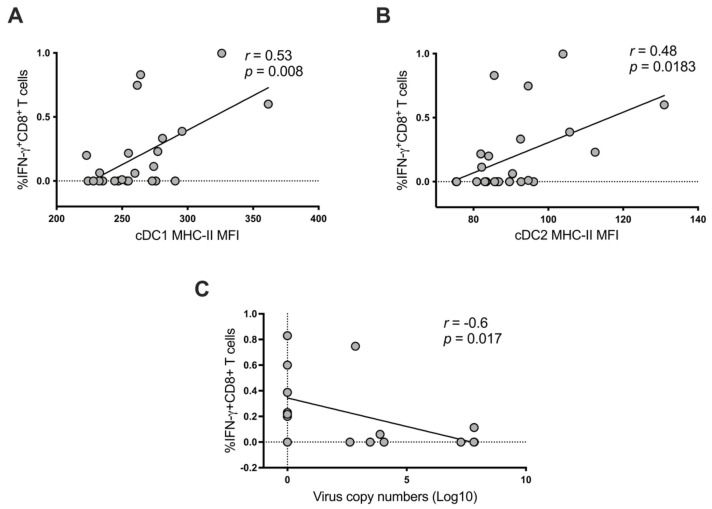
Correlation between CSFV-specific IFN-γ^+^ CD8^+^ T cells, viral loads and activation status of cDC1 and cDC2 in tonsils. (**A**) Correlation between CSFV-specific IFN-γ^+^ CD8^+^ T cells and MHC-II expression levels of cDC1 cells (*n* = 24). (**B**) Correlation between CSFV-specific IFN-γ^+^ CD8^+^ T cells and MHC-II expression levels of cDC2 in pigs (*n* = 24). (**C**) Correlation between CSFV-specific IFN-γ^+^ CD8^+^ T cells and viral loads at 36 and 90 h post-infection, when viral loads were detected in the tonsil (*n* = 16). Statistics were evaluated by a Spearman correlation test (Spearman r and *p*-values shown in graph).

Finally, to interrogate the importance of the observed changes, we carried out a correlation analysis between the IFN-γ^+^ CD8^+^ T cell responses, viral copy numbers and the modulation of MHC-s II expression on cDC subsets in C-strain vaccinated pigs using a Spearman correlation test. There was a positive correlation between the MHC-II expression of cDC1 cells and the percentage of CSFV-specific IFN-γ^+^ CD8^+^ T cell responses and a similar, albeit less significant correlation observed between MHC-II expression by cDC2 cells and the percentage of CSFV-specific IFN-γ^+^ CD8^+^ T cell responses (Figure 6A,B), indicating that the activation of both cDC subsets was directly correlated with the appearance of IFN-γ^+^ CD8^+^ T cells. Consequently, we tested the impact of the number of IFN-γ^+^ CD8^+^ T cells on the viral load. To do so, data from 36 and 90 h p.i. were used since these were the only timepoints with detectable viral copy numbers. Indeed, a negative correlation (s = −0.6, *p* = 0.017) between CSFV-specific IFN-γ^+^ CD8^+^ T cells and the viral load was identified (Figure 6C). These results indicate that the activation of tonsillar cDC1 and cDC2 subsets of C-strain vaccinated pigs may contribute to the early expansion of local CSFV-specific IFN-γ^+^ CD8^+^ T cells which could assist with the control of challenge infection.

## 3. Discussion

The role of the innate immune system in initiating an immune response to control viral infections is crucial and the ability of DC in particular to activate naïve T cells is well established. There is therefore considerable selective pressure for viruses to develop strategies to evade or counteract the development of a protective immune response generated by the host. In the case of CSFV, it is known that the virus targets myeloid cells such as macrophages and CD172a^+^ DC [10,11,12,13,14,15] resulting in the secretion of high levels of pro-inflammatory cytokines, generating not only a detrimental environment for the initiation of an adaptive immune response, but also an increased pathology and potentially more adverse clinical signs [23]. Conversely, the live attenuated C-strain vaccine is highly efficacious and rapidly establishes a robust T cell and subsequent antibody response [7,8]. Since the tonsil is a primary target for CSFV [18], it was considered the most appropriate tissue in the present study to best evaluate changes in both the innate and adaptive local immune responses at early timepoints following oronasal inoculation. Using a pathogenic CSFV strain in parallel to the attenuated CSFV C-strain enabled us to evaluate the role of tonsillar myeloid cells in the activation of the adaptive immune response and analyse the events in relation to viral replication.

During the initial 90 h post-infection, there were no significant clinical signs, although viral replication was detected in the tonsil (Figure 1) confirming previous studies [32]. The fact that the virulent Alfort-187 strain replicated more vigorously was expected and was in line with our in vitro experience regarding the kinetics of Alfort-187 and C-strain replication in permissive cell lines. Together these data validated our approach and confirmed significant, local differences between the C-strain and Alfort-187 infections. Taking the tropism of CSFV for myeloid cells into account, we next evaluated the kinetics, activation and infection status of porcine tonsillar myeloid cells as defined recently [28]. CSFV inoculation appeared to modulate the abundance of myeloid cells in the tonsils with some discernable differences emerging between the C-strain and Alfort-187 infections. For example, infection with Alfort-187 led to a trend in the reduction in the frequency of both the cDC1 and pDC, while upon C-strain inoculation their frequency increased, significantly in the case of cDC1 cells. More so, upon C-strain inoculation, cDC1 cells also temporarily displayed a significantly increased level of MHC-II expression (Figure 3), which could correlate with an elevated activation of these cells by the vaccine strain. We also observed fluctuations in cell frequency relative to the control group and both the CD14^+^ monocytic cells and CD163^+^ macrophages increased slightly over time, potentially indicating an overall increased pro-inflammatory response.

A limitation of the current study is its exploratory nature. Accordingly, only a limited number of animals could be used here, following the 3R principles. We therefore could not identify biologically significant events that require a greater resolution to determine.

A role for pDC as modulators of CSFV pathogenesis and drivers of the CSFV immune response has been described previously [15,33,34]. In accordance with this, we saw a trend for elevated pDC numbers in tonsils isolated from C-strain inoculated pigs, compared to Alfort-187 infection (Figure 2). There also appeared to be a greater number of pDC infected with Alfort-187 compared to the C-strain (Figure 4). It is thus tempting to assume that infection with the pathogenic strain compromises the pDC function, while the C-strain in associated, bystander cells stimulated the correct pDC function. These results are consistent with previous work which confirmed the association of CSFV with IFN-α positive cells (pDC) in the tonsil [15]. It would align with the fact that pDC are strongly stimulated by infected bystander cells [35] and conversely suggests that infection with pDC leads to an aberrant immune reaction, which the CSFV infection is characterised by highly increased IFN-α serum levels [23].

One interesting finding in our study was the emergence of a hitherto undetected population of CD14^+^CD163^+^ myeloid cells (Figure 2) that were also the most susceptible to CSFV infection (Figure 4), not discriminating between Alfort-187 and C-strain therein. The nature and function of this additional cell population remains enigmatic. The phenotype of this cell population determinates it to be of the myeloid linage, potentially monocytes upregulating CD163, as shown numerous times in humans and pigs, including when CD163 was first characterised [36,37,38]. CD163 is also a marker of macrophages, has been previously used to characterise such in pig tonsils [28] and is known to render pig monocytes/macrophages susceptible to PRRSV [39], but has not been associated with CSFV replication. Recently, however, it has also been proposed that a cDC3 population exists with the hallmark of CD14 and CD163 co-expression [40]. This population is currently subject to further investigation and characterisation in the field of DC biology [41], but as yet has not been identified in pigs. Clearly, further investigation is required to determine the nature of this population. Nevertheless, it is tempting to speculate that this putative cDC3 population plays an important role in determining the outcome of CSFV infection and vaccination, given that their kinetics parallel the infection dynamics in the tonsil (compare Figure 1, Figure 2 and Figure 4) more than it associates with either Alfort-187 or C-strain inoculation.

It has previously been postulated that cDC (identified as CD172a^+^ cells) were infected with CSFV [15]. Using a higher resolution, we are able now to refine this for the first phase of CSFV infection, showing that neither cDC1 nor cDC2 become infected, but rather this novel CD14^+^CD163^+^ population becomes infected, which also expresses CD172a as a broad myeloid cell marker. It will be interesting to see whether the known association of CSFV with myeloid cells in other organs including the spleen, kidney, lung, liver and the intestine [12,13,16,42,43,44] also associates strongly with a CD14^+^CD163^+^CD172a^+^ population as shown for tonsils in the present study.

An early stimulation of the immune response by the attenuated C-strain—or the lack thereof by a pathogenic CSFV strain—has been demonstrated little thus far. The infection in macrophages and the enigmatic CD14^+^CD163^+^ cells with the pathogenic Alfort-187 strain at 36 h post-infection might bias a pro-inflammatory environment, which could prevent an efficient T cell stimulation. In a recent study, a downregulation of co-stimulatory molecules and an upregulation of inhibitory/regulatory receptors across DC was observed by transcriptome analysis at a similar time point (42 h post-infection) and it was suggested that these alterations in T cell targeting receptors might explain the defects in the adaptive immune response [45].

Furthermore, our results indicate an early immune activation, with a trend towards an early increase in IFN-γ producing, virus specific CD8^+^ CTL following C-strain infection, which aligned with the changes seen in cDC1 cells in C-strain vaccinated pigs (Figure 1 and Figure 2) and their capacity to cross-present antigens [46,47]. Indeed, cross-presentation would be important in this scenario since our data clearly show that cDC1 cells did not become infected by CSFV (Figure 4). The emergence of a local CD8^+^ T cell response in the tonsil following C-strain vaccination is supported by a recent transcriptional analysis of tonsil tissue. Here, tissue from C-strain vaccinated pigs demonstrated an upregulation of *eomes*, a transcription factor, known to play a key role in the differentiation of effector CD8 T cells [48].

While it is unclear to what extent this CD8^+^ T cell population contributes toward the high efficacy of this vaccine, it is tempting to speculate that they support the early anti-viral response that limits early virus replication and dissemination, but further investigation is needed to confirm this. However, others have demonstrated an immediate primary CD8 T cell response which occurs within 24 h post-vaccination with a vaccinia virus construct [42]. Furthermore, T cells clustered around virus-infected DC in the draining lymph nodes of mice only 6 h following challenge with vaccinia virus, again suggesting an early interaction between the DC and T cell populations [49]. Accordingly, it is perhaps not surprising that our data showed a strong inverse correlation between the frequency of virus specific CD8 T cells in the tonsil and levels of virus (Figure 6).

In summary, the preliminary findings described here indicate a putative role for resident tonsillar cDC subsets in the induction of CD8^+^ T cell responses in vivo following C-strain inoculation and support the use of the C-strain as an effective vaccine to control CSF. We also identified a novel myeloid cell population that is highly susceptible to CSFV infection. This population may be critical in modulating the immune response during CSFV infection in the tonsil. The relative importance of this compared to other myeloid cells populations requires further investigation to ascertain their roles, both for improving our understanding of the immune mechanisms that afford the high efficacy of the C-strain vaccine, but also for harnessing the immunostimulatory function of such cells to improve vaccines for CSFV and other porcine viruses.

## 4. Materials and Methods

### 4.1. Animals, Viruses and Study Design

36 Large White/Landrace crossbreed pigs of 10 weeks of age were randomly assigned to 9 groups of 4, ensuring the mean weight was equal between groups. At day 0, 4 of the groups were inoculated with the CSFV strain Alfort-187, while 4 groups were inoculated with the C-strain *Riems*. One group was left untreated as a control, receiving only the equal amount of carrier (mock) solution. Both the CSFV Alfort-187 and C-strain were prepared using PK15 cells. C-strain *Riems* was grown from a stock originally supplied by Riemser Arzneimittel GmbH (Riems, Germany). CSFV Alfort-187 and C-strain were harvested by the freeze-thawing of PK15 cells and were clarified by centrifugation before titration on PK15 cells. The mock solution was prepared in the same manner from uninfected PK15 cells cultures.

For each virus strain, groups of pigs (*n* = 4) were euthanised after 9, 18, 36 and 90 h post-infection. The group of mock inoculated animals (*n* = 4) was used as a control group and euthanised at 9 h post-infection. The pigs were housed in high containment facilities at APHA and pigs inoculated with the different CSFV strains were kept in separate rooms. Intranasal inoculation was conducted by administration of 2ml of virus (or mock) per animal using a mucosal atomisation device MAD—300 (Teleflex, Beaconsfield, UK) and dividing the volume equally between nostrils. Both the C-strain and Alfort-187 inoculum contained 1 × 10^5^ TCID_50_. The titre of the Alfort-187 and C-strain inoculum was confirmed by back titration on PK15 cells.

### 4.2. Clinical Monitoring of Pigs

Pigs were monitored twice a day and at each euthanasia timepoint. Pigs were clinically scored for liveliness, body tension, body shape, breathing, walking, skin, eye/conjunctiva, appetite and defecation. Observations were scored as: 0 (normal), 1 (slightly altered), 2 (distinct clinical signs), 3 (significantly altered), as previously described [50]. A clinical score for each animal was assigned at each euthanasia timepoint. Rectal temperatures were monitored by thermometer readings daily and prior to euthanasia.

### 4.3. Assessment of Peripheral Blood Mononuclear Cell Counts

5 mL of blood was taken pre-inoculation and then again prior to euthanasia. Blood was collected in ethylenediaminetetraacetic acid (EDTA) containing Vacutainers (BD Biosciences, Oxford, UK) before peripheral blood mononuclear cells (PBMC) were separated via Histopaque 1.077 (Sigma-Aldrich, Poole, UK). PBMC were stained with anti-CD45-FITC (Clone K252.1E4, Bio-Rad Antibodies, Kidlington, UK) and resuspended in 5 mL of PBS before all cells were counted using a MACSQuant flow cytometer (Miltenyi Biotec, Bisley, UK).

### 4.4. Detection of CSFV in Blood and Tonsils

The total RNA was extracted from 140 μL of EDTA treated blood or 1 × 10^6^ tonsil cells using the QIAamp Viral RNA kit (Qiagen, Crawley, UK) according to the manufacturer’s instructions. The number of CSFV genome copies in each sample was determined by RT-qPCR with primers CDF-192-R and CSF100-R (600 nM) and with a FAM labelled CSFV probe (200 nM) [51], using the One-Step Superscript Platinum kit (Life Technologies, Thermo Fisher Scientific, Warrington, UK) as described previously [50]. RNA transcribed from the plasmid pCRXLV234-6 (containing the 5′UTR from the Alfort-187 strain) was used as standard to determine the CSFV genome copy numbers.

### 4.5. Enrichment of Tonsillar Myeloid Cells by Negative Selection Using Lymphocyte-Specific Antibodies

The isolation of mononuclear cells from the tonsil was performed as previously described [28]. Briefly, mononuclear cells were incubated for 15 min at RT with the lymphocyte-specific antibodies, anti-CD3 (clone 8E6), anti-CD8a (PT36A) (both from Washington State University Monoclonal Antibody Centre, Pullman, WA, USA), anti-CD21 (clone BB6-11C9.6, Southern Biotech, Cambridge Bioscience, Cambridge, UK) and anti-IgM (Clone K52 1C3, Bio-Rad) antibodies in PBS with 2% FBS (FACS buffer). After incubation, the cells were washed twice in a FACS buffer and then incubated with anti-mouse IgG microbeads (Miltenyi Biotec) for 15 min at room temperature. The cells were then washed twice in a FACS buffer and resuspended in a FACS buffer supplemented with 2 mM EDTA (MACS buffer). Cells were then applied to LD columns held in a MidiMACS magnet (Miltenyi Biotec). The flow-through, containing the lymphocyte depleted fraction and the two 1 mL washes with MACS buffer were collected in 15 mL tubes. The cells were washed twice with a FACS buffer and resuspended in 10 mL of RPMI-1640 (Life Technologies) supplemented with 10% FBS (Autogen Bioclear, Calne, UK), (cRPMI), before cell counting by volumetric flow cytometry using a MACSQuant Analyzer (Miltenyi Biotec).

### 4.6. Assessment of Myeloid Cell Frequency and Virus Infection

The evaluation of myeloid cell frequencies in isolated tonsil cells was performed by flow cytometry. Tonsil cells were surface stained in three consecutive steps. Cells were initially incubated with anti-lineage antibodies against CD3, CD8a, CD21 and IgM, (all mouse IgG1 antibodies) and Zombie Aqua viability dye (Biolegend, London, UK) to detect live cells. In addition, anti-CD4-PerCP-Cy5.5 (clone 72-12-4; BD Pharmingen, Oxford, UK), anti-CD14 PE-Texas Red (clone Tuk4; IgG2a Invitrogen, Thermo Fisher Scientific, Paisley, UK), anti-MHC class II-DR (clone 2E9/13; Bio-Rad) conjugated with Zenon anti-mouse IgG2b PE (Life Technologies, Thermo Fisher Scientific) and anti-CADM1-biotin (TSLC1/Syn-CAM, Clone 3E1; IgY, Caltag Medsystems Ltd., Buckingham, UK) were added. After washing twice, lineage positive cells were identified by staining with a secondary anti-mouse IgG1 Brilliant Violet 421 (Clone RMG1-1; BioLegend) while Streptavidin Brilliant Violet 605 (BioLegend) was added for CADM1 detection. Finally, cells were stained with anti-CD172a FITC (clone BL1H7; Bio-Rad) and anti-CD163 (clone 2A10/11; Bio-Rad) Zenon-labelled with APC or APC-AF750 (Life Technologies). All antibody incubations were at 4 °C for 20 min. To assess the cells for CSFV infection, the same protocol was applied with the addition of intracellular staining using a mouse antibody WH303 (APHA, Addlestone, UK) specific to the E2 protein of CSFV. Briefly, once the staining of the surface antigens was complete, the cells were washed twice with a FACS buffer and incubated with 100 μL of BD Cytofix/Cytoperm (BD Pharmingen) for 20 min at 4 °C before washing twice with PermWash (BD Biosciences). Cells were then labelled with 5 μL of WH303 Zenon-labelled with APC and incubated for a further 30 min at 4 °C. Finally, the cells were washed twice with PermWash (BD Biosciences) and resuspended in 100 μL of a FACS buffer and stored in the dark at 4 °C until the flow cytometric analysis on a LSRII Fortessa flow cytometer (BD Biosciences). Data analysis and compensation were conducted using Kaluza Software (Beckman Coulter, High Wycombe, UK) and the infection of the different cell subsets was evaluated by calculating the percentage of infected cells within each population.

### 4.7. Evaluation of T Cell Responses

Tonsil mononuclear cells were seeded at a concentration of 1 × 10^6^ cells/well in 100 μL of cRPMI in round-bottom 96-well plates (Costar, Sigma-Aldrich, Poole, UK). After seeding, CSFV Alfort-187 was added at a multiplicity of infection (MOI) of 9. An equivalent volume of mock-infected cell supernatant was used as a negative control and pokeweed mitogen (Sigma-Aldrich) at a concentration of 10 μg/mL was used as a positive control. The plates were cultured for 17 h (5% CO_2_, 37 °C) before the addition of BD GolgiPlug (BD Bioscience) at a final concentration of 0.2 μg/mL and incubation at 37 °C for a further 6 h. Cells were then washed twice with a FACS buffer and incubated for 10 min at room temperature with 5 μL of Live Dead Fixable Near IR dead stain (ThermoFisher), anti-CD8 PE (clone 76-2-11, BD Biosciences) and anti-CD4 PerCP-Cy5.5 (clone 74-12-4, BD Biosciences). After surface staining, the cells were washed twice with a FACS buffer and incubated with 200 μL of BD Cytofix/Cytoperm (BD Pharmingen) for 20 min at 4 °C. After washing twice with BD Perm/Wash (BD Pharmingen) the cells were incubated for a further 30 min at 4 °C with anti-IFN-γ AF647 (clone P2G10, BD Biosciences) and anti-TNF BV421 (clone MAb 11, Biolegend) diluted in BD Perm/Wash. After washing the cells twice with BD Perm/Wash (BD Pharmingen) they were finally resuspended in 200 μL FACS buffer before analysis on a MACS Quant Analyzer (Miltenyi Biotec). Data analysis and compensation were conducted using MACS Quant Software (Miltenyi Biotec).

### 4.8. Statistical Analysis

Statistical methods and the number of samples/animals used are detailed in the figure legends. GraphPad Prism 7.0 (GraphPad software, La Jolla, CA, USA) was used for the analysis of data sets.

## Data Availability

The data presented in this study are available on request from the corresponding author.

## Supplementary Materials

The following are available online at https://www.mdpi.com/article/10.3390/ijms22168795/s1, Figure S1. Gating strategy for assessment of infection in distinct tonsil myeloid cell populations in vivo during early stages of infection, Figure S2. Gating strategy for evaluation of T cell responses IFN-g and TNF-a in the porcine tonsil.
