# Supplementary material for: Activation of Dendritic Cells in Tonsils Is Associated with CD8 T Cell Responses following Vaccination with Live Attenuated Classical Swine Fever Virus
Source: Int J Mol Sci. 2021 Aug 16;22(16):8795. doi: 10.3390/ijms22168795 (PMC8395915; doi:10.3390/ijms22168795)
Supplement: Supplementary file 1 [file ijms-22-08795-s001.zip › ijms-1242412-supplementary.pdf]

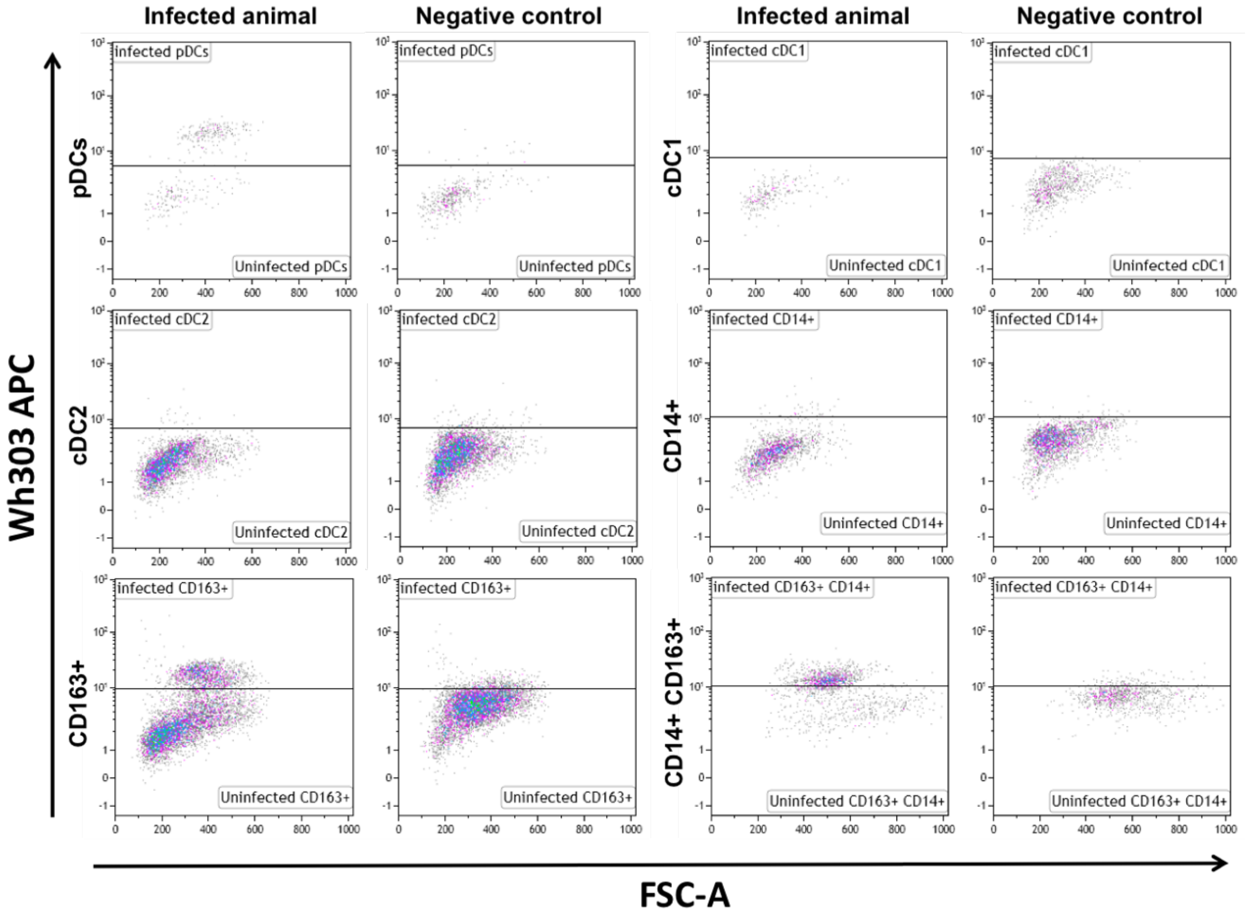


**Supplementary Figure S1 – Gating strategy for assessment of infection in distinct tonsil myeloid cell populations *in vivo* during early stages of infection.** Porcine myeloid cells were isolated from tonsil mononuclear cells by depletion of cells expressing lineage markers, namely, CD3, CD8$\alpha$, CD21 and IgM. After enrichment, cells were stained with Live Dead, CD4, CD14, CD172a, CD163, CADM1 and MHC-II followed by intracellular staining of CSFV using the monoclonal antibody Wh303. Gating strategy to define each of the myeloid cell subsets described in by our group (Soldevila et al., 2018) was applied and infection levels were evaluated on each subset by WH303 positivity. Comparison of infected vs uninfected animal is shown for each myeloid cell subset with the addition of $CD14^{+}CD163^{+}$ cells.

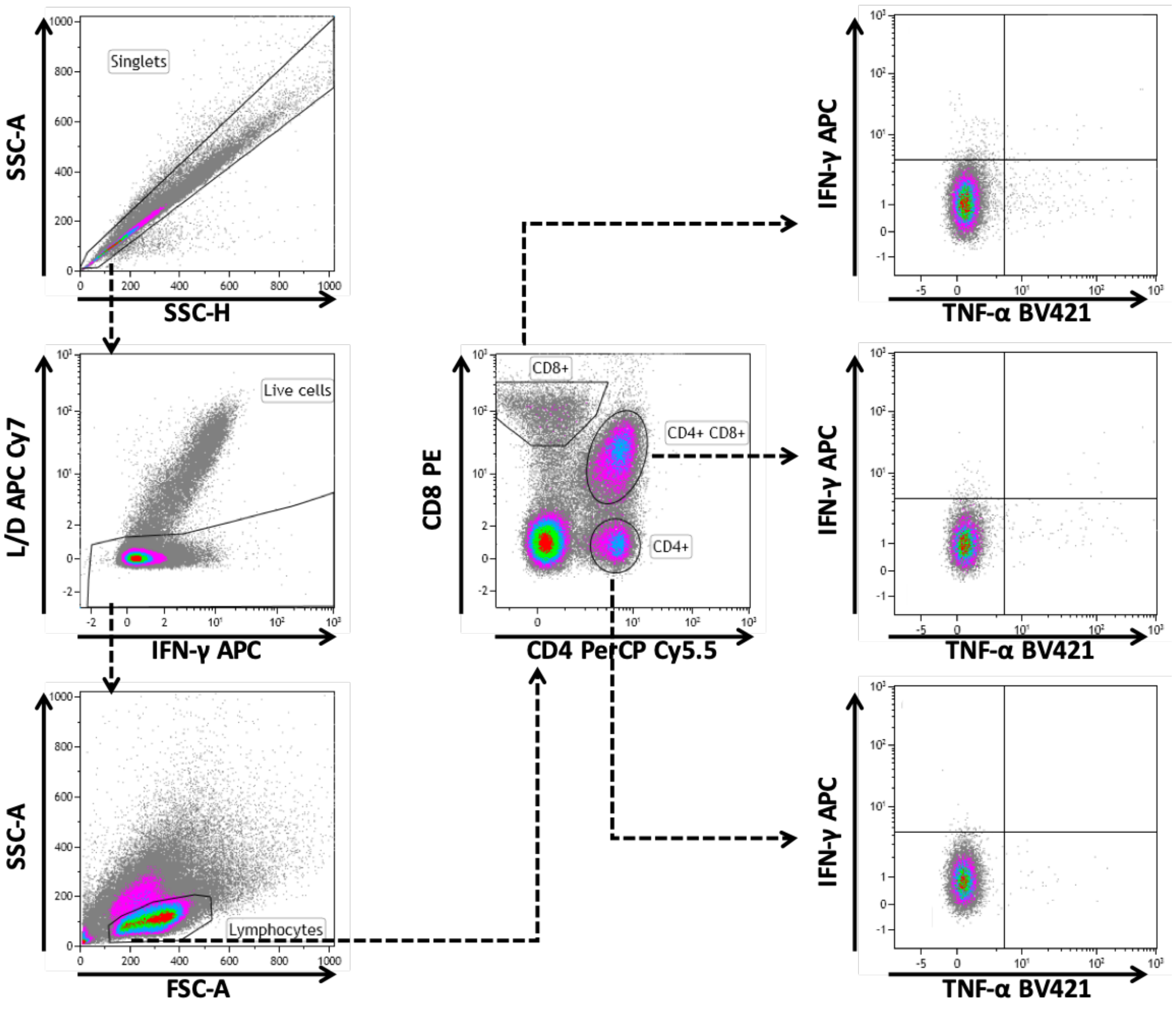


**Supplementary Figure S2 – Gating strategy for evaluation of T cell responses IFN-γ and TNF-α in the porcine tonsil.** Tonsil mononuclear cells obtained by Ficoll gradient were seeded in round bottom 96-well plates and stimulated with Alfort 187 for 17 hours before addition of GulgiPlug for a further 6 hours. After incubation cells were surface-stained with Live/Dead, anti-CD4, anti-CD8α followed by intracellular staining of IFN-γ and TNF-α. Dot plots from one of the animals in the study representing gating strategy with doublet discrimination, followed by dead cell exclusion and lymphocyte selection by physical characteristics are shown on the left side. Central dot plot was used to define CD4 T cells, CD8 T cells and double positive CD4 CD8 T cells respectively, followed by the assessment of percentage of cells of each populations expressing either IFN-γ and TNF-α or both.
